# Cost-effectiveness of dobutamine stress cardiac magnetic resonance imaging in stable coronary artery disease - a post-hoc analysis

**DOI:** 10.1186/1532-429X-15-S1-O8

**Published:** 2013-01-30

**Authors:** Sebastian Kelle, George Petrov, Sarah Kern, Eckart Fleck, Ernst Wellnhofer

**Affiliations:** 1 Internal Medicine/Cardiology, German Heart Institute Berlin, Berlin, Germany

## Background

Dobutamine stress cardiac magnetic resonance imaging (DCMR) was not an established test for coronary ischemia in 2003/2004. Meanwhile, long-term outcome has demonstrated a strong prognostic value of negative DCMR testing (Kelle et al. JACC CVI 2011). However, only limited data are as yet available about reimbursement for and cost-effectiveness of DCMR in patients with stable coronary artery disease (CAD).

## Methods

An electronic patient information system (PIS) archive allowed the selection of a matched control group of patients undergoing cardiac catheterization (CA) without prior DCMR. Our institution takes part in the German Diagnose Related Groups (DRG) reimbursement scheme and has transmitted micro cost calculations for all in-hospital stays of patients to the InEK database since 2003. The study cohort of this "natural experiment" 2003-2004 comprises 706 patients with suspected coronary artery disease (284 female, mean age 60 ± 10). A group of patients with DCMR guided treatment strategy (DCMR) (N= 264; CA: N=65; no CA: N=199) was matched to a control group with CA indicated by standard evaluation (SECA) (N=442).

## Results

Framingham risk score did not differ significantly between groups (7.7 ± 2.8 vs. 8.1±3.2). Outcome was better in DCMR than in SECA. This trend was borderline significant in the above sample. An evaluation in a larger sample (N=924; 270 SCMR; 654 SECA) comprising additional patients from this period without cost data demonstrated a significantly (P<0.006 log-rank test) superior outcome in DCMR that persisted even after adjustment for cardiovascular risk and revascularization. Median incremental costs related to hospital stays were 12.713 € ± 43.664 € (median 4864 €) in the SECA and 4104 € ± 4454 € (median 3387 €) in the DCMR group. Differences were located on the ward, intensive care unit and operation room but not in the cath-lab. All types of costs particularly human resource costs were elevated in the SECA group. Incremental cost-effectiveness ratio was calculated as 822 € per survival. Sensitivity analysis demonstrated that at least 750 € /per patient are saved by DCMR. Death, PCI, CABG, and hospital admissions were 6%, 1%, 1%, and 35% in the DCMR and 11%, 34%, 20%, and 97% in the SECA group over 8 years.

## Conclusions

DCMR is cost-effective in competition with SECA even if a reimbursement of 750€ for each DCMR examination is calculated. This natural experiment allows the simulation of detailed realistic cost-effectiveness scenarios for health technology assessment of DCMR.

## Funding

none

